# Global Comparative Law?^†^

**DOI:** 10.1093/ojls/gqae038

**Published:** 2024-11-16

**Authors:** Fernanda Pirie

**Keywords:** Comparative law, expanding the field, new perspectives

## Abstract

*The Cambridge Handbook of Comparative Law*, edited by Matthias Siems and Po Jen Yap, continues recent calls to expand the field of comparative law. By including authors drawn from all parts of the world, it presents ‘new perspectives’ on the field. This wide geographic remit proves successful as a way of moving beyond traditional ‘families’ and doctrinal topics. The contributors raise new themes for comparison, many related to public law and processes of change. But this, in turn, raises questions about the purposes of expanding the field. The volume largely concerns the laws and legal issues of modern states, and the authors do not venture far into history. Nor do they consider the alternatives offered by religious and traditional legal systems or forms of non-state ordering. I suggest that these subjects could productively expand the field even further, raising more theoretical questions about what law is and does.

## 1. Introduction

Since the early 2000s, comparative law scholars have repeatedly made calls to expand their field, both in terms of subjects and methods. Siems and Yap’s ‘new handbook for comparative law in a global context’ aims, likewise, to extend scholarship beyond ‘traditional’ functional methods and private law topics.^1^ Bringing together scholars ‘drawn from all parts of the world’ to write on topics that relate to different geographic regions, the editors seek to present ‘new perspectives’ on the field.^2^ Latin America, Sub-Saharan Africa, the MENA region (Middle East and North Africa), South Asia and East Asia all feature among the chapters, along with others on ‘law beyond the state’.

This method for expanding the field of comparative law is productive. Several chapters highlight new topics for comparative exploration. But the editors’ approach also raises more general questions: what is the object of expanding the field? What sorts of issues can the comparison of diverse examples address? Is the best way to do this by seeking ‘new perspectives’ from authors based in different places? And if the goal is expansion, how much further might the field extend and to what ends? Given the recent trends, this volume provides a good opportunity and good material with which to consider these issues.

In this review article, I first consider the content of the volume and the arrangement of its chapters.^3^ I ask what the contributions reveal about the breadth of the field, actual and possible, and its subject matter. I suggest that the collection highlights issues that emerge from the exploration of laws and legal systems that are quite distant, in terms of historical origins and social and political contexts. However, it stops short of embracing the very disparate forms of law that have emerged in many historic and non-state contexts. The great religious legal systems of the past and the legal traditions of indigenous people and premodern contexts are not directly considered. So I go on to ask whether scholars can and should extend their inquiries further in such directions.

I conclude that scholarship should continue to be exploratory, embracing a range of examples, approaches and methods. Rather than being defined by its methods, the field should aspire to incorporate different types of laws and legal systems in order to address different sorts of questions. Scholarship can range from the close consideration of laws that address similar issues within cognate systems (what might be called more traditional forms of comparative law) to the comparison of more distant examples, as found in this volume. These point to consideration of context and change. Going yet further, comparison of the very different legal systems that emerged in ancient civilisations and among indigenous groups is likely to raise more theoretical and general issues about the nature and role of laws in societies.

The current volume, it could be said, points the way from micro to macro issues, from doctrine to context and from substance to process, although scholarship might go further in all these directions.

## 2. Academic Context

Over the past 25 years, many comparative lawyers have advocated diversifying their subjects of study. These have included Legrand and Munday, Reimann and Zimmermann, and Örücü and Nelken.^4^ Others have called for new methodological approaches.^5^ Several handbooks, edited volumes and encyclopedias highlight the different topics that might be compared and the different parts of the world that might be studied.^6^ As the editors of the current volume remark,^7^ these handbooks have advanced the study of comparative law by going beyond the classic functional approach and private law topics, applying different methods and considering different areas of law.

Anticipating the enthusiasm for expansion, Reimann sounded a note of caution in 2002, warning of what he called a ‘laissez-faire’ attitude to comparison and calling for a distinct ‘canon’.^8^ Twenty years later, Husa has described the field as ‘a jungle’.^9^ But he, along with Matthias Siems, one of the editors of the current volume, have consistently promoted expansion.^10^ Quoting Cotterrell’s call to ‘prolong puzzlement’, Siems advocates ‘postmodern’, socio-legal, numerical and empirical methods, embracing what others have seen as the challenge of comparing ‘foreign laws’.^11^ ‘Global comparative law’, he argues, should be an ‘open subject’, encompassing interdisciplinary approaches and considering influences that shape such laws, including their genealogies, common structures and shared features.^12^ Universalities and singularities among them, he points out, may be shaped by such things as ‘broad intellectual or theoretical trends and movements, societal developments, and the political climate’.^13^

This vision for ‘global comparison’ indicates the study of process, as much as substance, examining how laws develop, converge and diverge, and the multiple factors that influence change. Siems also emphasises the importance of considering context. He calls for ‘interdisciplinarity and cosmopolitanism’ in order to steer comparative law in a more contextual direction, as he puts it, enhanced by research in other comparative disciplines.^14^ Where such moves do and could lead, in terms of the questions that comparatists can address, are issues that I address in this review.

## 3. The volume

The editors, Siems and Yap, express their aims in terms of opening up a ‘diversity of perspectives’. Their objective is ‘to reflect on the different ways we understand the “law” and how it operates in practice’.^15^ To do so, they have invited scholars from different parts of the world to bring ‘new perspectives’ to the field. It is evident that many of the topics the authors have chosen reflect regional dynamics and issues that particularly concern those working in different parts of the world. Ultimately, the editors explain, their object is ‘to find commonalities that bridge the divide between legal traditions or geographies’.^16^

The chapters are grouped into four parts. These concern ‘methods of comparative law’, ‘legal families and geographical comparisons’, ‘central themes’ and ‘law beyond the state’. In this way, it could be said, the volume addresses the *how* of comparative law (methods), the *what* (families) and the *why* (themes), as well as offering a *vision* for the field (to extend ‘beyond the state’). In fact, as one might expect, there are many overlaps between the sections. Different themes are discussed throughout the volume. In what follows, I discuss the various aspects of the field that the chapters represent, considering each section in turn.

### A. Methods

Comparative law should, it might seem, be a field defined by its methodology. Not surprisingly, discussions of methods are prominent in most handbooks and edited collections.^17^ Other disciplines, particularly in the social sciences, offer any number of methodologies that could be embraced by comparative lawyers. Yet the functionalist approach, which has dominated the field since the 1970s, is still prominent, and calls to pursue other methods have not resulted in distinctly new bodies of scholarship. In 2023, the outgoing editors of the American Journal of Comparative Law described their efforts to widen the journal’s scholarship, noting a decrease in ‘classical’ private law comparison, but beyond the rise of comparative constitutional law, they did not identify any other prominent themes, directions or methodologies.^18^ Does this cast doubt on the centrality of method as a means of either defining or expanding the field? Some have suggested that different disciplinary approaches might also provide the basis for an expanded field, although the editors have chosen not to highlight this in the current volume.^19^

The nine chapters in this section of the volume discuss a variety of methods and approaches, old and new. Many of them are innovative and could be (or are currently being) applied in legal (and other) scholarship more generally. These include historical-jurisprudential methods (Halpérin), linguistic approaches (Biel), qualitative fieldwork (Mahy and others), new institutional economics (Sabiiti), quantitative empirical methods (Siems) and machine learning (Ho and others).^20^

These chapters provide a positive complement to much earlier scholarship, critically surveyed by Husa in the first chapter in the section. ‘Traditional methods’, he explains, have tended to pursue their inquiries within narrower confines: concentrating on contrasts between civil and common law systems, private law topics and doctrine. Several scholars have emphasised the difficulties of comparison, particularly when considering non-Western and non-state laws.^21^ By exploring a variety of methods, the chapters in this volume provide a positive and welcome complement to these debates, as well as to the ‘radical scepticism’ expressed by Legrand about the comparative consideration of different sorts of laws.^22^

The result is somewhat eclectic, however: some of the authors link their approaches to scholarship in broader disciplinary fields, such as linguistics, sociology and economics. On the other hand, this section does not aim to be comprehensive and to highlight all, or even the main, comparative methods that could be used in legal scholarship. Rather, it gives a sense of new possibilities within an expanding field, one that is not limited by traditional concerns and objectives. Presumably other methods could be added. These chapters point to an exploratory approach towards comparison, then, one that takes advantage of new directions in wider scholarship and allows authors with particular methodological and disciplinary expertise to explain how their work can contribute to the field. This highlights what I consider to be one of the main strengths of the volume: that the editors have allowed individual contributors to explore new (and old) possibilities.

On the other hand, important though methods are and rigorous though comparatists need to be, particularly when employing specialist techniques, it is not clear that any particular method or disciplinary approach is leading, or will lead, to a well-defined body of new scholarship. Reimann’s concerns about ‘laissez-faire’ approaches resonate here. In any event, the issue of methodology should not eclipse questions about the broader objectives of any exercise in comparison. ‘Why compare?’ is surely a prior question. Does the idea of legal families and geographies suggest a better foundation for an expanding field?

### B. Families and Geography

This section contains an interesting and thought-provoking group of chapters. The title harks back to classic approaches in comparative law, which defined the world’s legal systems in terms of ‘families’. The numerous problems with traditional classifications are discussed by Halpérin in this volume.^23^ But other chapters in this section consider new areas of commonality, largely found in geographical groupings. These include the laws of former Soviet states (Shirvindt), the jurisdictions of Latin America (Zuloaga and de Valdés), the Middle East and North Africa (Elsaman), South Asia (Abeyratne) and Sub-Saharan Africa (Fombad).

Laws and legal systems have influenced and borrowed ideas and doctrines from one another over time, and still do. As a result, laws with similar features may be quite distant in time and space, so it makes sense to seek out commonalities among disparate systems.^24^ But the chapters in this volume also draw attention to the importance of geographical context and to the historic, political, economic and religious factors that influence the shape that laws take. For example, in accounting for both similarities and differences, the authors point to the experience of Soviet domination (Shirvindt), to a history of colonial trajectories and processes of modernisation (Zuloaga and de Valdés, Elsaman) and to contemporary regional trends towards development and legal reform (Elsaman, Fombad, Zuloaga and de Valdés). At the same time, they avoid simple country definitions, recognising that commonalities often transcend national borders.

In some quarters, classification has been seen as a prerequisite for comparison. It may help the researcher select the ‘jurisdictions’ to study, as Zuloaga and de Valdés put it.^25^ But there is a danger that to classify encourages us to assume similarity more than difference. This may lead to a form of comparison that starts by selecting ‘jurisdictions’ based on type, rather than allowing the juxtaposition of cases to lead to insights into commonalities and difference, as well as similarities that cross typological boundaries. At the very least, classifications may overlap in interesting ways. Indeed, it could be said that several of the chapters in the volume, in both this and later sections, do just that, for example discussing regional patterns, but also highlighting differences among systems of private law in Latin America (Caffera and others), forms of public law in East Asia (Yap) and dynamics of legal pluralism in Africa (Ordor and others). Given this legal complexity, the traditional aim of classifying laws and legal families is surely outdated. On the other hand, embracing a wide geographic remit is clearly productive, as this volume demonstrates.

At the same time, the volume might have included chapters that focused on the processes through which laws develop and diverge. How do commonalities emerge and what accounts for difference? Obvious candidates include colonialism in its various forms, modernity and self-conscious attempts to reform legal and political systems, a desire to be seen to meet global standards, and the influence of international bodies and technical developments, as well as internal issues, such as the need to recognise the interests and traditions of indigenous groups. All of these are illustrated by chapters in this volume, as well as other scholarship.^26^

In this spirit, Halpérin advocates the comparative study of legal change as a way to move away from a focus on ‘legal families’ and ‘national spirit’. His approach would see legal change as the outcome of multiple factors, social, political and economic, and not primarily in the adoption or ‘transplant’ of legal concepts, principles and ideas. The question of change, of course, relates to well-worn debates within comparative law on ‘legal transplants’, which are considered by several chapters in the following section of the volume.

### C. Themes

Watson, in his famous work on legal transplants, emphasised the influence of legal ideas when they are transplanted into new contexts. His arguments were provocative and compelling, even if, as many scholars have pointed out, he was wrong to downplay so radically the influence of context.^27^ The analysis of content, process and context must all come together to explain legal change. The resulting complexity is exemplified by several chapters in this section of the volume. Cohn, for example, examines the ways in which public law doctrines emerge and are transported around the world, emphasising the fact that such processes are ongoing and involve multiple participants. Yap contributes to the debates on constitutions and constitutionalism by considering convergences and divergences in constitutional jurisprudence among different jurisdictions within East Asia, specifically the constraints on political activities exercised by courts. Caffera and others consider legal transplants in the development of private law in Latin America. All exemplify the benefits of studying the multiple factors involved in such processes.

But apart from change and convergence, this section hardly identifies central themes for an expanding field. Other chapters concern topics that are rather niche: the development of company law and shareholder protection in Turkey (Kandemir); the effects of conflict in Afghanistan (Mobashar and Rahimi). These are interesting in their own right, but their themes are not exactly central. Other topics, old and new, recur throughout the volume. Comparisons of what might, broadly, be called public law topics are found in all sections of the volume, at least matching those that focus on private law: Abeyratne describes a singular style of constitutionalism, which seems to be emerging in South Asia; Zuloaga and de Valdés describe regional changes in public law in Latin America; and Fombad compares programmes for legal reform in Sub-Saharan Africa. These and other authors consider the contextual factors that account for the shape that laws take, while chapters in the last section of the volume consider the importance of regional organisations in the development of business law (Cuyvers), compare approaches to the conflict of laws (Nishitani), describe different attempts to recognise indigenous values in Africa (Diala) and highlight the globalisation of legal education (Cheng-Han and others)

The volume presents a variety of legal subjects, then, suggesting that the field is diversifying too much to identify any unifying themes. Nevertheless, the editors might have noted a trend to study topics in public, as much as private, law and to consider context more than doctrine.

### D. Beyond the state

Similar issues are evident in chapters of the last section of the volume, which includes the study of laws that transcend state boundaries. The importance of geopolitical and economic context is very evident here. For example, Rachad and Mendes, in their chapter on international law, discuss the different ways in which states approach their obligations under international laws, contrasting the United States, EU countries and those of Latin America. They trace the ways in which states are more or less open to human rights and environmental laws, treaties and principles. In the case of human rights, they relate these differences to national policies on foreign relations, and in the case of environmental law, to differences in the ways in which such law is conceived. Their consideration of borrowing and change is complemented by Daly’s discussion of cross-border judicial dialogue as a factor in legal development, surely an important and neglected topic in the analysis of legal change. Further issues emerge in other chapters in this section.

## 4. A Global Vision for Comparative Law?

This volume encompasses a diversity of themes, then, as well as geographies and methods. Does it suggest a body of scholarship that transcends, in a coherent and productive way, the traditional focus on legal families and national legal systems, and on doctrinal and private law? Does it point the way to more ‘global’ comparisons, between legal systems that have developed in quite different contexts and been influenced by quite different factors?

In this regard, two questions arise. First, how and why might comparative legal scholarship go beyond traditional topics? This question has already been addressed in multiple ways in previous literature, but does the volume point to any new answers? I suggest that a broad geographic remit is productive in this regard, as well as the emphasis on process and change. Second, does the volume give the sense of a broader or ‘global’ field for comparative inquiry, or does it indicate one that is diverse and fragmenting? Is there a danger that the field will fail to be more than the sum of its parts, no more than a set of disparate studies of different legal topics, as Reimann warned?^28^ Even if the scholarship has expanded far beyond anything that could be called a ‘canon’, does the current volume point to a set of themes and questions that it is worth exploring in systematic ways?

As regards the volume’s broad geographic remit, I have already suggested that when considering laws on a global scale, scholarship is likely to encompass macro more than micro issues. It may also exted to public more than private law and to move from the comparison of doctrine to consideration of institutions, networks, and social and political context.

Much traditional comparative legal scholarship has considered the doctrines of legal systems that are, in many ways, similar. These have typically been those of the Global North, and the main contrasts have been between common law and civil law systems. The aims of such work have generally been to explore how different jurisdictions approach similar legal issues, themselves relating to common social problems.^29^ This broadly functional approach works well for the detailed comparison of doctrine among closely cognate legal systems.^30^ Scholars may, as a result, be able to suggest different, and better, legal solutions to common problems. More broadly, they might seek to better understand and generate new thinking on doctrinal issues. For notable recent examples, we could point to work on the relationship between criminal and tort laws; on the nature of fiduciary duties; and on the issue of vicarious liability.^31^

Another strand of comparative law scholarship has been concerned with legal transplants, convergence and divergence. Historical research on the contextual and cultural factors explaining similarity and difference includes Whitman’s study of laws that reflect attitudes towards honour and dignity in Germany and the United States.^32^ Scholars with more of a policy orientation, meanwhile, have promoted or criticised projects of legal convergence, European harmonisation and processes of globalisation.^33^

Detailed comparison at the level of doctrine, the basis of much of this scholarship, is only, however, possible when considering legal systems that are similar in their structures, aims and contexts. Doctrine can only fruitfully be compared in the case of laws produced by states that are structurally alike, in which law makers hold analogous political values and pursue similar social objectives. If the social and political context is very different and the shape of the legal systems has been affected by a multiplicity of different factors, productive forms of comparison need to take context, in all its complexity, into account. This leads to consideration of broader issues. These are exemplified by many of the chapters in the volume, which consider reasons for convergence and divergence, the influence of historical factors, but also the emergence of regional dynamics and transnational developments.^34^

This indicates that comparison of more distant examples is likely to raise broad questions, such as how laws come about, how and why they take particular forms, what roles they are expected to play in societies and how they are shaped by social, political and economic factors. This is to focus on process more than substance, and origins more than effects. All of this suggests moving away from comparison based on issues defined in doctrinal terms and recognising the plurality of influences on the forms that laws take. In practical terms, it means searching for cross-cutting commonalities and differences among instances of law, and allowing that they might emerge in surprising contexts and take unexpected forms, influenced by multiple factors.

A lingering urge to typologise must, then, be resisted. To take one example, law and legal practice in contemporary China has been influenced by ancient Confucian values, as Ngoc Bui explains in his chapter in this volume. But they have also been shaped by the region’s imperial and socialist histories, by the civilian grounding of Japanese law, on which the contemporary system was based, and by the ongoing commercial and technological objectives of the modern state, to name just a few.^35^ Ngoc Bui’s chapter draws attention to the influence of Confucian ideas in large areas of the Far East, a useful comparative exercise, but the Chinese legal system (and doubtless others in that region) cannot be classified under any single heading.

Two sorts of themes could be said to emerge from this volume, then. First, the emphasis on different geographies points to consideration of what might be called macro issues, including public laws and their objectives, the roles of lawyers and legal institutions, the relationship between courts and law makers, the pursuit of social justice, attempts to recognise indigenous values, and so on. This might be grounded in the exploratory search for commonalities and differences, leading to the consideration of context, as long advocated by proponents of an expanding field. Second, comparison can highlight the reasons for convergence and divergence. This indicates the analysis of process as much as substance and the multiple influences that determine the shape that laws take, both historic and contemporary.

In this way, it could be said, the volume points the way to scholarship that can paint a picture of a dynamic global legal landscape, in which numerous different jurisdictions are in a continuing process of change and development.

## 6. Further Expanding the Field?

The editors’ implicit vision for the field of comparative law is positive and expansive, then. It is also exploratory. Their strategy has been to seek contributions from authors based all over the world, with the aim of gathering different perspectives.^36^ This opens the field to topics, issues and methods developed in different academic contexts, by scholars whose work is inevitably shaped by their own institutional and political concerns. Those who have confronted issues that arise uniquely, or particularly acutely, in a particular geographic region are likely to have thought more deeply about certain themes—the effects of colonialism and its aftermath, wars and other crises, development projects, legal pluralism, the interests of indigenous people, and so on. Local perspectives on these issues are valuable.

The limitations of this approach also need to be recognised, however. Academia forms a set of global networks. Academics speak common languages and address common themes. Legal scholars, from whatever parts of the world, form a transnational elite, connected through publications, conferences, online discussions and careers that often span many institutions and countries. Moreover, the training of most legal scholars is based on the study of national legal systems. Apart from the contributions on international and transnational laws, most of the chapters in this volume, even those that concern indigenous norms, focus on developments within state legal systems. Should the field extend beyond such topics, not least in response to the call for ‘decoloniality’ made by two chapters in the volume? In the last part of this review, I consider how comparative scholars might pursue such an agenda by considering other forms of non-state law, including historic, religious and traditional.

### A. Decoloniality

The volume includes two chapters on ‘decolonial’ approaches (Dedek, Merino). Implicitly, these advocate expanding the field beyond the parameters of the modern state and the legacies of European colonialism that have shaped the laws of the Global North. Like other work in this vein, they are largely critical and focus on the discipline and its history. Dedek describes the colonial backdrop to the 1900 Paris Conference, at which the discipline of comparative law is said to have been born. Elsewhere, Salaymeh and Michaels describe the foundations of the discipline in 19th-century methods developed in the Global North.^37^ Such critical approaches lead to questions about where scholarship should go next. Salaymeh and Michaels advocate ‘pluriversality’ as a means to integrate non-formal law and recognise non-state law. Comparative legal history, they say, should focus on law ‘prior to modernity’. The implication is that (comparative legal) scholars should take into account ‘other’ laws, legal systems and perspectives, those not tainted by (European) colonialism. But whose and how?

Merino, in this volume, suggests exploring the experiences of scholars from indigenous groups in colonised countries. He proposes working with legal activists and ‘norm entrepreneurs’. This would lead to an explicitly political agenda: ‘to engage directly with social actors on the ground through decolonial methodologies … engaging with and supporting their political agendas’.^38^ Such scholarship would focus on a narrow section of the population, however, those pursuing such political projects. These are, by definition, people who operate in a world shaped by colonialism and its aftermath, who must speak the languages of the (erstwhile) colonisers and who are often educated in institutions founded on their principles. And should we necessarily champion their projects—what if ‘traditional’ laws embody values that have come to seem outdated elsewhere? Such communities should, of course, be taken seriously, and their views and activities are important topics for study, leading to insights into the impacts of colonial histories and alternative legal strategies, among other things. However, for real alternatives to modern state laws, we must take seriously other forms of law, such as those that flourished prior to the disruptions of European colonialism (although they may well have been affected by, or instruments in, other forms of colonialism).^39^ Such laws can be found in the past and in non-state contexts, but also in the very different legal systems that remain powerful phenomena in the contemporary world, as I describe in the following section.

For all the editors’ attempts to bring together different perspectives, then, and despite the inclusion of chapters on ‘decoloniality’, the subjects of this volume remain overwhelmingly modern state law. Why and whether this should define the field of comparative law is possibly the most valuable question raised by the volume, albeit one it does not answer.

### B. Non-state Laws: Historical, Indigenous and Private Orders

How might comparative lawyers encompass a wider range of laws, then, and should they do so? To gain truly different perspectives, we have to make the effort to understand how people in very different social and political contexts, speaking other languages and undergoing distinct sorts of education, organise their societies. How do they address and understand problems of violence and disorder, and create, use, adopt or reject forms of law? Exploring such questions could mean, to take an obvious example, considering the laws developed and applied by Islamic scholars, who are still prolific today. Some comparatists have considered Islamic law.^40^ There is a huge (non-comparative) literature on Islamic law, both its historical forms, which were radically different from modern state law, and also developments in the contemporary world. There is extensive debate, for example, on the nature and significance of attempts by modern states to base their legal systems on *shariʿa* principles and whether the result is a form of ‘Islamic law’ or not.^41^ Then there are studies of modern shari‘a courts and the continuing role and influence of traditional scholars.^42^

Comparative lawyers have rarely considered these or other historic systems, despite calls to do so.^43^ They have rarely delved into the extensive scholarship on the laws of the Near Eastern, Jewish, Hindu and Chinese traditions, even those of classical Rome. Multiple smaller traditions also flourished throughout the world during the last two millennia, in such places as Ireland, Armenia and Tibet.^44^ Yet, even the more expansive collections and encyclopedias on comparative law rarely include such examples. Comparatists like Bussani and Mattei^45^ have emphasised the importance of history, but few authors in the current volume delve far into the past. In the following section, I discuss what comparisons among such examples might reveal.

Seeking different perspectives could also mean considering the social contexts (semi-autonomous social fields) in which alternatives to state laws emerge, often called forms of ‘private ordering’. Such studies form a distinct field, as discussed by Grisel in a remarkable historic and ethnographic study on a community of fishers in southern France.^46^ Grisel analyses the laws and norms of the fishers’ *Prud’homie de Pêche* by comparison with many other examples, including the norms developed by diamond traders in New York, the Sicilian mafia, lobster fisheries in Maine, trade in Mexican California, international rating agencies and prison gangs. As he points out, many of the authors of these studies are economists, but there is more that can be learned by taking historic and sociological perspectives on the development and effects of such norms, which offer clear comparative potential. Grisel, for example, assesses the benefits and weaknesses of these forms of private governance as alternatives to regulation by law, critically assessing some of the assumptions regularly made by economists about their ‘efficiency’.

Why have comparatists been reluctant to embrace the possibilities offered by such studies? One of the issues, particularly presented by historic and indigenous laws, is that much of this is work for specialist scholars, principally historians, anthropologists and those with specialist language skills. To consider comparatively the long and influential legal traditions of the Chinese and Hindu worlds, which flourished from the second centuries (BCE and CE, respectively), as well as the Islamic and Roman traditions, non-specialists would have to rely on the work of classical China scholars and experts in Sanskrit, classical Arabic and Latin, as well as historians and archaeologists of these regions. Similarly, any comparatist who wanted to consider the laws created by historic mercantile networks, village codes, the rules of guilds and the work of historic legal scholars would need to turn to historians or, for contemporary equivalents, anthropologists trained in ethnographic methods. Even to consider Islamic law in the modern world, expertise is needed to understand the work of the highly specialist legal scholars and the long, complex and intellectually sophisticated traditions in which they work.

But why not enter into conversation with such specialists? The Max Planck Institute on Comparative and International Private Law has, since 2009, incorporated a research group on Islamic law. Networks and collaborations between different scholars can lead to insights from comparison of empirical examples widely spread in time and space.^47^ Others profitably use secondary sources.^48^

### C. An Expanding Field?

What, then, would such conversations and collaborations achieve? Simply highlighting difference leads to an appreciation of diversity. It might also present alternatives to the dominant model of state law and the concerns of the contemporary world. It is surely valuable, in itself, to put modern laws into historic context and recognise the sophisticated civilisations and laws that were largely denigrated and eclipsed by the forces of colonialism and modernity. But such laws also offer more direct comparative potential. Considering systems that are less cognate means asking different questions, not just about how laws address their objectives, but also what those objectives are or were. It raises questions about who makes laws and what issues they are attempting to address, as well as their effects and influence on wider society.

When cases are distant, comparison, I have argued, needs to take account of context. This is even more so in the case of laws that are rooted in very different social and political times and places, where a multiplicity of different factors have shaped the objectives of the law makers and those who use their texts. As Huxley puts it, ‘we have to understand and sympathise with the scholarly community that preserved the texts and the interpretive traditions that applied them’.^49^ Simply to understand such laws, it is necessary to consider the broader political objectives of the law makers, as well as the underlying. generally unstated, social and moral values of the local context. As Honoré explains, in a study on the common law, laws respond to social tensions and objectives not by setting out basic moral values and social principles (do not kill or steal, keep your promises, be fair in your dealings, and so on), but by specifying how those values are to be realised, what sorts of penalties or sanctions should be imposed, what constitute exceptions and defences, and so on.^50^ Knowledge of context is generally needed simply to understand what such laws mean.^51^

By the same token, comparison among more distant examples means drawing upon empirical evidence about the dominant social (and religious) values, political structures and economic constraints. For example, early laws—those produced in Mesopotamia, Israel, India, China and Rome—largely addressed similar topics (killing, injury, theft, sexual misconduct, orphans, slavery).^52^ But the ways they did so reflected their origins and the contexts in which the authors worked: judicial practices in the courts of Babylon were brought together and codified for an ascendant warlord; tribal traditions in Israel were written down by scholars trained in Near Eastern traditions within a priestly project to unify the community of Israelites; the Hindu Brahmins who were the authors of the Dharmaśāstras were confirming a caste hierarchy which reflected their view of the *dharma*, the cosmological foundation of the world; in China, laws were developed by rulers to impose order through discipline and punishment; and in early republican Rome, the Twelve Tables were created at the instigation of an assembly of citizens seeking social justice.^53^ Of course, these extremely brief synopses are highly simplistic and raise as many questions as they address. But such examples indicate that any comparative work has to take into account a multitude of social, political, religious and economic factors. This presents obvious challenges to comparison, but it also points to what might be gained.

Comparison among such examples inevitably leads to consideration of what can be considered truly macro issues, such as the nature of the law makers and the role of law in their societies. Asking who was responsible for the forms that laws took, for example, we might consider whether the authors were specialist scholars, their forms of education and whether they borrowed from other traditions. How, we could also ask, were the laws developed, reproduced, copied and commented upon, and in what contexts, why and by whom? Then there are questions about the role that such laws were intended to and did play, inviting obvious comparisons between religious laws, in which priests and muftis had authority, and those in which authority was asserted by political leaders, such as the emperors of China and Rome. These are the sorts of ‘big questions’ which, as Huxley argues, distinguish comparatists from stamp collectors.^54^

Comparisons of wide-ranging examples can, in these ways, address broad theoretical questions about the forms that laws take and their roles in societies, as well as who makes law and why. Often it is commonalities among very different forms of law or legal practice that are most suggestive, indicating recurrent social patterns, although surprising differences can be just as revealing. To give just two examples, Don Davis, a specialist in classical Hindu law, has traced parallels between the legal opinions of the Brahmins and the work of Roman and Islamic jurists. This raises questions about the role of scholarship in the development of legal systems, which might be further investigated by considering other examples.^55^ Meanwhile, the anthropologist Judith Scheele found analogies between the village laws she documented in highland Algeria and laws made by Spanish villagers in the 16th century. But a surprising absence of similar laws in analogous village communities suggests that the reasons for law making in such circumstances must have been more than functional.^56^ Again, further questions arise about both these and other examples.

Any form of comparison amongst such very different examples requires an exploratory approach. It also means being open-ended in the questions asked, rather than trying to specify in advance which aspects of law to examine and compare. Scholars have to leave behind traditional themes and topics for comparison, to embrace context and to ask about change.

Previous attempts to compare legal systems on a global scale have, I would suggest, been marred by a lingering concern to classify. Patrick Glenn considered a wide range of legal ‘traditions’.^57^ He emphasised their changing nature but, nevertheless, described them as conveying legal ‘ideas’ or ‘information’, thus forming a set of distinct and discrete ‘traditions’. This suggests comparison at the level of substance and doctrine, rather than process and context, and it draws attention away from the law makers, their objectives and the multitude of factors that must have influenced the ways in which such laws developed.^58^

Within the field of socio-legal studies (or ‘law and society’ scholarship), some scholars have compared examples concerning the ways in which law operates. Questions about the administration of justice have, for example, been addressed comparatively by Mirjan Damaska.^59^ Other studies were brought together in an earlier volume on comparison in socio-legal studies, as well as chapters in other collections.^60^ Why, indeed, should comparative legal scholarship remain distinct from comparative socio-legal studies? But if comparative law is to retain a distinct focus, I suggest, it needs to consider questions about the forms and shape that laws take and what influences them, as much as the social effects of law and legal processes, important though these are.

### D. Limits?

Are there, nevertheless, limits to legal comparison? How, for example, is law to be identified for comparative purposes? This question becomes acute when scholars consider indigenous norms and non-state orders. A huge body of literature has focused on the concept of ‘legal pluralism’, which many, including myself, consider expands the category of law too far.^61^ Halpérin is one contributor to address the issue directly in the current volume. This is also an issue for those who advocate ‘decoloniality’. The answer, as I have suggested elsewhere, is that it may be necessary to work with more clearly definable concepts, like ‘legalism’ or ‘private ordering’. There is always a risk that studies of law will shade into studies of government, regulation, new technologies and other related issues. But this may not, in itself, be such a problem. It is for legal scholars to identify the distinctly legal (or legalistic) elements among such examples and to highlight and explore their comparative potential.

Is there also a problem with fragmentation? Husa suggests that comparative legal scholarship is dividing into the comparative study of particular topics, comparative constitutional law being one of the most notable.^62^ These can generate distinct debates, often associated with their own journals. But this should not prevent more general comparison across different examples. A broad field might encompass a number of sub-fields, as well as promoting more wide-ranging forms of comparison.

Is it also necessary to draw a sharp divide between studies that are explicitly comparative and those that simply concern ‘different’ forms of law? The current editors of the AJCL invite studies of ‘legal systems, cultures, and traditions *other than those of the US*’ (emphasis added). These contributions need not, apparently, be explicitly comparative.^63^ But publishing them in a single journal surely serves to bring such studies into conversation with each other and with work on more familiar legal systems. It invites readers to consider their comparative potential. Even simple juxtaposition can lead to reflection on similarities and differences. This is one way to promote the exploratory approach that, I have suggested, is a positive feature of the volume under review.

The alternative would be either to ignore the very different forms of law that have existed throughout the world or to regard them as topics for distinct studies, debates and journals. Embracing their comparative potential, on the other hand, is the best way to develop a field of truly global legal scholarship. The questions that juxtaposition and comparison can address are varied, I have argued here, as are the methods that might be employed. The resulting field will also be far more diverse than that characterised by traditional comparative methods and topics. But surely this is to be welcomed. The aspiration to a global comparative perspective has the potential to generate truly original ideas.

## 7. Conclusion

The current volume answers calls for diversity in the field of comparative law, which have been made repeatedly over the last two decades. It demonstrates that the expansion of geographic focus can be productive, pointing to the consideration of different sorts of law, as they emerge in different contexts, without preconceptions as to the ‘families’ or legal topics that should ground the comparison. But the fact that it has not proved so easy for comparatists to move beyond the state and embrace what is truly different indicates that there is much more that might be done in this regard.

Comparative legal scholars have hardly dipped their toes into the vast and lightly charted waters of non-state laws. Here, they might engage with scholarship from other disciplines, whether textual studies on Islamic jurisprudence or ethnographies of private orders. Lawyers, I have suggested, can profitably enter into conversation with legal and other scholars, who offer expertise in laws found in different parts of the world. Together, they may offer insight into issues that bear fruitful comparison across large distances.

Considering comparative law scholarship on a spectrum, we might identify three types. First, legal systems that are close to one another in social and political context lend themselves to comparison of doctrinal issues, to topics in private law and to pursuit of the goal of improvement. This is what might be called ‘traditional’ comparative law. Nothing I have said in this review should be taken as denying the importance of such studies, and this field (or these diversifying fields) will surely continue to produce valuable scholarship.

When comparing, secondly, more distant forms of law, fruitful comparison is more likely to take into account contextual factors, including historic and regional developments, political structures, economic goals and other constraints. It also demands more open-ended questions and the exploration of commonalities and differences that may emerge in surprising places. Comparison at this level invites us to consider process, development, reasons for change and convergence, origins, contexts and the objectives of the laws, as much as their effects. These are themes that run throughout the current volume. This scholarship can aspire to build a picture of a dynamic global legal landscape.

Comparing, thirdly, what is really different—religious, historic and traditional systems—the questions become more theoretical, about the nature of law and its social role. Methodologically, such comparison benefits from multidisciplinary expertise and the use of secondary sources. This calls for work that encompasses the studies of multiple scholars from a variety of disciplinary backgrounds: comparing the results of existing studies and engaging in collaborative projects. Building a really broad field of comparative law need not be an endeavour for legal scholars working alone.

This volume charts a course into the second form of comparison, although it hardly touches on the third. But together, these three strands of comparative scholarship can address, in uniquely productive ways, different aspects of law: what it is, what it does, how it develops and what role it plays in different societies. This is to answer the most important question: why should legal scholars compare? Some comparative studies enhance distinct bodies of doctrinal legal scholarship and should continue to do so. But there must also be room for scholarship that is more exploratory, for the comparison of laws separated by long times and large distances. The more expansive the comparison, the more distant the forms of law, the more likely it is to open up new issues for consideration. It will draw attention to emerging forms of law, to legal techniques and their deployment, to the different sorts of people who create, use and influence laws, and to the contexts in which they are shaped. And it is surely valuable, in its own right, to consider the alternative forms of law that do exist, and have existed, beyond the state. This can give us an invaluable sense of the different things that law is, has been and can be. The field may be diverse and the scholarship exploratory, but expansion can be embraced, if scholars are prepared to confront and address challenging questions about what law is and does.

## Footnotes

1
*Cambridge Handbook* 2.

2 ibid xvii.

3 Inevitably, I will not be able to do justice to each contribution, so I will concentrate on those that seem most relevant to the broader discussion in this review. Equally, I cannot attempt to do justice to the whole field of comparative law, so, again, I will concentrate on examples of scholarship that best illustrate the trends and possibilities I foreground in this article.

4 Pierre Legrand and Roderick Munday (eds), *Comparative Legal Studies: Traditions and Traditions* (CUP 2003) include chapters on a particularly wide range of subjects. Örücü, in her chapter in that volume (‘Comparatists and Extraordinary Places’), calls for studies on parts of the world (Eastern Europe, Turkey, Hong Kong) characterised by diversity, multiculturalism, legal pluralism and the dynamics of legal transpositions. Mathias Reimann and Reinhard Zimmermann (eds), *The Oxford Handbook of Comparative Law* (1st edn, OUP 2006). Esin Örücü and David Nelken, *Comparative Law: A Handbook* (Hart Publishing 2007) is presented as an explicit attempt to widen the field beyond private law and legal families (v–vi) and to encompass what the editors see as contemporary debates.

5 PG Monateri, *Methods of Comparative Law* (Edward Elgar 2012); PG Monateri, *Advanced Introduction to Comparative Legal Methods* (Edward Elgar 2022); Geoffrey Samuel, *An Introduction to Comparative Law Theory and Method* (Hart Publishing 2014); Jaakko Husa, *A New Introduction to Comparative Law* (Hart Publishing 2015); Jaakko Husa, *Introduction to Comparative Law* (Hart Publishing 2023).

6 Mathias Reimann and Reinhard Zimmermann (eds), *The Oxford Handbook of Comparative Law* (2nd edn OUP 2019) includes chapters on the ways in which the study of comparative law itself have been pursued in different countries; Örücü and Nelken (n 4); Mauro Bussani and Ugo Mattei (eds), *The Cambridge Companion to Comparative Law* (CUP 2012) describe different legal subjects, traditions and disciplinary approaches. The vast encyclopedia of JM Smits and others (eds), *Elgar Encyclopedia of Comparative Law* (first published 2006, Edward Elgar 2023) incorporates 225 chapters by authors from 50 different countries.

7
*Cambridge Handbook* 2.

8 Matthias Reimann, ‘The Progress and Failure of Comparative Law in the Second Half of the Twentieth Century’ (2002) 50 AJCL 671.

9 Jaakko Husa, ‘Comparative Law’s Pyrrhic Victory?’ (2023) 30 MJ 680, 681.

10 Matthias Siems, *Comparative Law* (first published 2014, CUP 2022).

11 ibid 139, 149–50, quoting Roger Cotterrell, ‘Comparative Law and Culture’ in Reimann and Zimmermann, 2nd edn (n 6) 721.

12 Siems (n 10) chs 14 and 15.

13 ibid 435 and 447, quoting Anne Peters and Heine Schwenk, ‘Comparative Law Beyond Post-modernism’ (2000) 49 ICLQ 800, 829.

14 Siems (n 10) 453. Advocates for the consideration of context are prominent in many other collections, especially Örücü and Nelken (n 4), as highlighted in a useful review by Reza Banaker, ‘Power, Culture and Method in Comparative Law’ (2009) 6 International Journal of Law in Context 69. Siems puts more emphasis on change and process.

15
*Cambridge Handbook* xvii.

16 ibid 11.

17 Notably, however, Reimann (n 8) 683–4 emphasised the importance of substance over method.

18 Helge Dedek and Franz Werro, ‘Changing of the Guard: Two “Gatekeepers” Bid Adieu’ (2023) 71 AJCL vii.

19
*Cambridge Handbook* 7. See Jaakko Husa, *Interdisciplinary Comparative Law: Rubbing Shoulders with the Neighbours or Standing Alone in a Crowd* (Edward Elgar 2022); Jaakko Husa, *A Research Agenda for Comparative Law* (Edward Elgar 2024). Siems (n 10) 413 refers to what he calls ‘the broad interdisciplinary approach to comparative law’ that forms the basis of his book.

20 Siems has consistently promoted the use of quantitative methods: ibid chs 8 and 9.

21 Esin Örücü, ‘Developing Comparative Law’ in Örücü and Nelken (n 4) 56–62.

22 Pierre Legrand, ‘The Impossibility of Legal Transplants’ (1997) 4 MJ 111; Pierre Legrand, ‘What “Legal Transplants”?’ in D Nelken and J Feest (eds), *Adapting Legal Culture* (Hart Publishing 2001) 55–70. The resulting debate is discussed by the editors in their Introduction (4–7). While Legrand’s scepticism is extreme, we must also acknowledge the great importance of his interventions in the field and their productive effects, not least the call to recognise context, as much as doctrine.

23 Jaakko Husa has also been a critic: ‘Classification of Legal Families Today: Is It Time for a Memorial Hymn?’ (2004) Revue Internationale de Droit Comparé 11. However, other scholars continue to promote forms of classification, eg Ugo Mattei, ‘The Legal Metaverse and Comparative Taxonomy: A Reappraisal’ (2023) 71 AJCL 900.

24 Commonalities apparent among legal systems with no apparent connections might be even more interesting, as I discuss in the last section.

25
*Cambridge Handbook* 311.

26 One might mention Upendra Baxi, ‘The Colonialist Heritage’ in Legrand and Munday (n 4) 46–75; Örücü, ‘Comparatists and Extraordinary Places’ (n 4) on processes of modernisation; and Riles’s study on Japan and its desire to be seen to meet global financial standards: Annelise Riles, *Collateral Knowledge: Legal Reasoning in Global Financial Markets* (University of Chicago Press 2011) ch 5.

27 Siems (n 10) 294–9 gives a good summary of the criticisms and resulting debates.

28 Reimann (n 8).

29 Konrad Zweigert and Heine Kötz, *An Introduction to Comparative Law* (3rd edn, T Weir tr, Clarendon Press 1998).

30 It might also be employed in more wide-ranging comparisons. See eg Petra Mahy, ‘The Functional Approach in Comparative Socio-Legal Research: Reflections Based on a Study of Plural Work Regulation in Australia and Indonesia’ (2016) 12 International Journal of Law in Context 420.

31 Matthew Dyson, *Explaining Tort and Crime: Legal Development across Laws and Legal Systems, 1850–2020* (CUP 2022); Lionel D Smith, *The Law of Loyalty* (OUP 2023); Paula Giliker, *Vicarious Liability in Tort: A Comparative Perspective* (CUP 2013).

32 James Whitman, ‘The Neo-Romantic Turn’ in Legrand and Munday (n 4) 312–44.

33 These issues are discussed and critically reviewed by Husa, *Introduction to Comparative Law* (n 5) 75–90. In the current volume, some of the authors do pursue what could be called more traditional objectives. The implicit goals of harmonisation and global governance are, for example, expressed in several chapters, including Nishitani on conflicts of laws. Fombad also asks questions about the success of continent-wide reforms in Africa. But here the issues, like those in most other chapters, are more macro, including the success of structural reform on the quality of justice and promotion of the rule of law, rather than detailed doctrinal developments.

34 Such themes have also been explored elsewhere, for example, to name just one, Virginia Harper Ho, ‘Beyond Transplant: A Network Innovation Model of Transnational Regulatory Change’ (2023) 71 AJCL 513.

35 Taisu Zhang, *The Laws and Economics of Confucianism: Kinship and Property in Pre-industrial China and England* (CUP 2017) offers an interesting historic and comparative account, albeit focused on just the last few centuries. For comparison between China and Japan, see Kentaro Matsubara, ‘East, East, and West: Comparative Law and the Historical Processes of Legal Interaction in China and Japan’(2018) 66 AJCL 769.

36 This is not exactly to explore the different ways in which we understand the ‘law’, as the editors put it in their preface (*Cambridge Handbook* xvii). That would suggest a more hermeneutic approach, teasing out understandings of what law is and does, and the study of ‘legal consciousness’—a term that has come to stand for a whole range of topics. As originally used by Ewick and Silbey, *The Common Place of Law: Stories from Everyday Life* (University of Chicago Press 1998), it captures the ways in which people (in that study, American citizens) understood what law was and did. Recognising the importance of such understandings could be said to underlie Legrand’s objection to the possibilities of legal transplants and the ‘neo-romantic’ turn discussed by Whitman (n 32). Such scholarship can be interesting and important, but comparative law needs a broader remit.

37 Lena Salaymeh and Ralph Michaels, ‘Decolonial Comparative law: a Conceptual Beginning’ (2022) 86 Rabels Zeitschrift fur Auslandisches und Internationales Privatrecht 166. Munshi describes trends to ‘decolonize’ comparative scholarship: Sherally Munshi, ‘Comparative Law and Decolonial Critique’ (2017) 65(suppl 1) AJCL 207.

38
*Cambridge Handbook* 425.

39 Chinese imperial regimes and Indian Brahmins, to name just two, used law in what could be regarded as projects of colonial or quasi-colonial expansion.

40 Siems (n 10) 123–6; Uwe Kischel, *Comparative Law* (A Hammel tr, OUP 2019) ch 10, who also nods towards Jewish and canon law. Other examples can be found in publications on comparative law: Mohammad Fadel, ‘Sovereignty, Territoriality, and Private International Law in Classical Muslim International Law’ (2023) 71 AJCL 853; Lena Salaymeh, ‘Comparing Islamic and International Laws of War: Orthodoxy, “Heresy,” and Secularization in the Category of Civilians’ (2021) 69 AJCL 136; Samy A Ayoub, *Law, Empire, and the Sultan: Ottoman Imperial Authority and Late Ḥanafī Jurisprudence* (OUP 2020); Khaled Abou El Fadl, ’The Islamic Legal Tradition’ in Bussani and Mattei (n 6) 295–312; Chibli Mallett, ‘Comparative Law and the Islamic (Middle Eastern) Legal Culture’ in Reimann and Zimmermann, 1st edn (n 4) 624–51. Mallett highlights the differences between classical Islamic and state law, but most of these studies are not primarily comparative.

41 Wael Hallaq, *An Introduction to Islamic Law* (CUP 2009) ch 10; Joseph Schacht, ‘Problems of Modern Islamic Legislation’ (1960) 12 Studia Islamica 99; Joseph Schacht, *An Introduction to Islamic Law* (Clarendon Press 1964) ch 15; Baudouin Dupret, ‘The Concept of Law: A Wittgenstinian Approach and Some Ethnomethodological Speciﬁcations’ in SP Donlan and others (eds), *Concepts of Law* (Ashgate 2014) 72, 74; Baudouin Dupret, *What Is the Sharia?* (Hurst 2018) ch 7.

42 See eg MK Masud, R Peters and DS Powers (eds), *Dispensing Justice in Islam: Qadis and Their Judgements* (Brill 2006); John R Bowen, ‘Anthropology and Islamic Law’ in AM Emon and R Ahmed (eds), *Oxford Handbook of Islamic Law* (OUP 2018); Morgan Clarke, ‘The Judge as Tragic Hero: Judicial Ethics in Lebanon’s Shari‘a Courts’ (2012) 39 American Ethnologist 106.

43 Werner Menski, ‘Beyond Europe’ in Örücü and Nelken (n 4) 189–216.

44 Notable exceptions are studies by scholars who do not, primarily, present themselves as comparatists: Andrew Huxley (ed), *Religion, Law, and Tradition: Comparative Studies in Religious Law* (Routledge 2002); Fernanda Pirie, *The Rule of Laws* (Profile 2021).

45 Bussani and Mattei (n 6) 4–5.

46 Florian Grisel, *The Limits of Private Governance: Norms and Rules in a Mediterranean Fishery* (Hart Publishing 2021).

47 Examples can be found in Huxley (n 44) and in a series of edited volumes: Paul Dresch and Hannah Skoda (eds), *Legalism: Anthropology and History* (OUP 2002); Fernanda Pirie and Judith Scheele (eds), *Legalism: Community and Justice* (OUP 2014); Paul Dresch and Judith Scheele (eds), *Legalism: Rules and Categories* (OUP 2015); Georgy Kantor, Tom Lambert and Hannah Skoda (eds), *Legalism: Property and Ownership* (OUP 2018).

48 Patrick Glenn, *Legal Traditions of the World* (5th edn, OUP 2014); Pirie, *The Rule of Laws* (n 44); Jean-Louis Halpérin, *Une histoire des droits dans le monde* (CNRS Editions 2023).

49 Huxley (n 44) 7.

50 AM Honoré, ‘Real Laws’ in P Hacker and J Raz (eds), *Law, Morality and Society* (OUP 1977).

51 This is why it is only possible to undertake detailed comparison of doctrine across similar social and political contexts.

52 For a translation of Hammurabi’s laws, see Martha Roth (ed), *Law Collections from Mesopotamia and Asia Minor* (Scholars Press 1995); the laws of Exodus (21:1–22.16) can be read in any version of the Old Testament; Manu’s Dharmaśāstra has been translated by Patrick Olivelle and Suman Olivelle, *Manu’s Code of Law* (OUP 2004) 123–54; the Tang code has been translated by Wallace Johnson, *The T’ang Code*, 2 vols (Princeton UP 1979–97); for an annotated translation and reconstruction of the Twelve Tables, see MH Crawford*, Roman Statutes*, vol 2 (Institute of Classical Studies, School of Advanced Study, University of London 1996).

53 For general background to these laws, see Pirie, *The Rule of Laws* (n 44).

54 Huxley (n 44) 7, citing William Ewald, ‘Comparative Jurisprudence (1): What Was It Like to Try a Rat?’ (1995) 143 U Pa L Rev 1889, 1892.

55 Donald R Davis, Jr, ‘Responsa in Hindu Law: Consultation and Lawmaking in Medieval India’ (2014) 3 Oxford Journal of Law and Religion:57.

56 Judith Scheele, *Village Matters: Knowledge, Politics, and Community in Kabylia (Algeria)* (James Currey 2009).

57 Glenn (n 48). Other scholars have followed suit: Bussani and Mattei (n 6) Part III.

58 Cogent criticisms have also been made by Annelise Riles, ‘Comparative Law and Socio-Legal Studies’ in Reimann and Zimmermann, 1st edn (n 4) 772–804, fn 98 and by Halpérin, *Cambridge Handbook* 46.

59 Mirjan Damaska, *The Faces of Justice and State Authority: A Comparative Approach to the Legal Process* (Yale UP 1991).

60 Fernanda Pirie, Agnieszka Kubal and Naomi Creutzfeldt (eds), *Comparative Socio-Legal Studies*, special issue (2016) 12(4) International Journal of Law in Context; Siems (n 10) ch 7.

61 Fernanda Pirie, ‘Beyond Pluralism: A Descriptive Approach to Non-state Law’ (2023) 14 Jurisprudence 1.

62 The comparative study of constitutions and constitutionalism could be said to form one of the (only) distinct trends in recent scholarship, and one that has been highly productive. See eg Tom Ginsburg and Rosalind Dixon (eds), *Research Handbook in Comparative Constitutional Law* (Edward Elgar 2011); Ran Hirschl, *Comparative Matters: The Renaissance of Comparative Constitutional Law* (OUP 2014).

63 See eg Benjamin Schonthal, ‘Buddhist Rules About Rules: Procedure and Process in the (Theravāda) Buddhist Legal System’ (2021) 69 AJCL 539.

